# Characterization of Protein Co-Localization and Histone Modifications at Enhancers Anchored in Chromatin Loops

**DOI:** 10.3390/biology15161331

**Published:** 2026-08-07

**Authors:** Wei Sheng, Yumin Nie

**Affiliations:** Department of Bioinformatics, School of Biomedical Engineering and Informatics, Nanjing Medical University, Nanjing 211166, China

**Keywords:** enhancer, chromatin loop, histone modification, A/B compartment, dual-function protein

## Abstract

Enhancers regulate genes over long genomic distances by physically looping to interact with promoters, a process highly dependent on specific protein recruitment and local histone modifications. We identified 1399, 1394, and 4063 enhancer-associated chromatin loops in GM12878, K562, and HepG2 cell lines, respectively, and found that dual-function proteins, characterized by both activating and repressing activities, preferentially localized at loop-anchored enhancers. Although protein co-localization patterns varied among cell types, CTCF, RAD21, SMC3, and ZNF143 consistently co-localized with one another at these enhancers. Furthermore, enhancer-associated loops localized primarily to the A compartment. Enhancers anchored in A-A loops showed greater enrichment of H3K4me1, H3K4me2, H3K9ac, and H3K27ac than those anchored in B-B loops. However, in K562 cells, enhancers anchored in B-B loops exhibited minor changes in active histone modification enrichment compared to their A-A counterparts, and even showed higher proportions of H3K4me2 and H3K9ac enrichment for enhancer–promoter loops. Nevertheless, across all three cell lines, A-A enhancer–promoter loops with both active anchors exhibited significantly higher target gene expression than other A-A loops and B-B loops. Our study suggests that enhancer-associated loop formation correlates with dual-function protein co-localization and active histone modification enrichment, which may collectively contribute to target gene transcription.

## Simple Summary

Enhancers are DNA sequences that can activate genes from a distance. They do this by forming physical loops to reach the genes they control. This process relies on specific proteins that bind to the DNA and chemical modifications that mark the surrounding DNA regions. In this study, we identified thousands of enhancer loops in three different cell types (GM12878, K562, and HepG2). We found that proteins with both activating and repressing functions were often present at the enhancers that anchor the loops. Although the overall patterns of these proteins varied between cell types, four specific proteins (CTCF, RAD21, SMC3, and ZNF143) consistently worked together at these sites. Furthermore, most enhancer loops were found in active regions of the genome, and enhancers in these active regions carried more activating chemical marks. Interestingly, in one cell type (K562), loops in inactive regions showed only small changes in these activating marks. Despite these differences, loops with both enhancer and promoter anchors active were always associated with higher gene activity across all cell types. Our findings suggest that enhancer loop formation is closely linked to the presence of these proteins and chemical marks, which may work together to regulate gene activity.

## 1. Introduction

Enhancers are non-coding regulatory sequences that activate target gene expression through transcription factor binding and the subsequent recruitment of architectural proteins, coactivators, chromatin remodelers and modifiers, and RNA polymerase II [1]. Enhancer activity is associated with local histone modifications. Inactive enhancers reside within a closed conformation, lacking transcription factor binding and characteristic histone modifications. By contrast, active enhancers are marked by the combination of H3K4me1 and H3K27ac, primed enhancers by H3K4me1 alone, and poised enhancers by H3K4me1 together with the repressive mark H3K27me3 [2,3].

Enhancers are often located distally from their target genes, and chromatin looping brings these distant regions into close spatial proximity to facilitate enhancer–promoter (E-P) interactions. Multiple proteins, including structural proteins, transcription factors and coactivators, are involved in chromatin loop formation. The DNA-binding protein CCCTC-binding factor (CTCF) and the cohesin complex are structural proteins that cooperate to establish chromatin loops and regulate gene expression via the loop extrusion model [4,5]. The ubiquitously expressed transcription factor Yin Yang 1 binds to active enhancers and promoter-proximal elements and dimerizes to promote E-P interactions [6]. Transcription factors containing intrinsically disordered regions (IDRs) within their activation domains, including the pluripotency factor OCT4 and the yeast transcriptional activator GCN4, form phase-separated condensates with the Mediator coactivator at super-enhancers to activate gene expression [7].

Chromatin loops are the finest and most fundamental structural units of three-dimensional chromosome organization. At a higher hierarchical level, the genome is compartmentalized into active (A) and inactive (B) compartments. A compartments generally contain transcribed genes and active histone modifications, whereas B compartments are highly condensed, gene-poor, and enriched for repressive histone modifications. Owing to phase separation, A and B compartments preferentially interact with regions of the same type, resulting in the characteristic plaid-like pattern observed on Hi-C contact maps [8]. Fine-scale compartment analysis revealed that active distal enhancers, characterized by H3K27ac in the absence of H3K27me3 and H3K9me3, predominantly localize to the A compartment, and E-P interactions display higher specificity than general A compartment association [9].

Enhancer activity is highly context-dependent, influenced by specific protein binding, the local distribution of histone modifications, and the three-dimensional organization of the genome. In order to investigate how enhancers are involved in chromatin loop formation and regulate target genes, we first identified enhancer-associated chromatin loops and analyzed the co-localization patterns of proteins at these loop-anchored enhancers in GM12878, K562, and HepG2 cell lines. Furthermore, we examined the enrichment of histone modifications at these enhancers to uncover the epigenomic features associated with the formation of chromatin loops. Finally, we identified E-P loops and investigated how histone modifications and chromatin looping are associated with target gene expression.

## 2. Materials and Methods

### 2.1. Data Source

A total of 49,672, 43,148, and 50,160 enhancers corresponding to the GM12878, K562, and HepG2 cell lines, respectively, were downloaded from the EnhancerAtlas2.0 database (http://www.singlecelldb.com/) (accessed on 25 May 2021) [10]. In addition, 51,381 promoter regions, defined as 2 kilobases (kb) upstream of transcription start sites of UCSC-annotated genes, were obtained from the UCSC Genome Table Browser (http://genome.ucsc.edu/cgi-bin/hgTables) (accessed on 24 October 2024). All genomic coordinates for these elements were mapped to the hg19 genome assembly.

Raw paired-end reads for in situ Hi-C experiments in GM12878, K562, and HepG2 cell lines were downloaded from the ENCODE portal (http://www.encodeproject.org/) (accessed on 16 June 2024) [11] under the accession numbers ENCSR410MDC, ENCSR545YBD, and ENCSR194SRI [12,13], respectively. Raw reads were first trimmed from their 3′ ends to the GATC restriction site, and then aligned to the hg19 human reference genome using the Bowtie2 software (v 2.5.0) [14]. Only uniquely mapped reads with a mapping quality greater than 10 were retained, and read pairs with identical ends were only considered once to eliminate PCR duplicates. Tag directories were further generated from the filtered reads using the HOMER program (v 5.1) [15] makeTagDirectory, followed by a second run with the parameters -removePEbg, -removeSelfLigation, and -removeSpikes 10,000 5 to remove small fragments, self-ligations and regions with abnormally high read density. Additionally, the parameter -restrictionSite GATC -both was applied to retain only read pairs whose both ends were located within the estimated fragment length from a restriction site. After read and read pair filtering, 715 million, 961 million, and 1162 million read pairs were obtained for constructing contact matrices in GM12878, K562, and HepG2 cells, respectively. Based on the definition of Hi-C map resolution as the smallest locus size at which at least 80% of loci have more than 1000 contacts [12], the maps for all three cell types reached 5-kb resolution, and chromatin loops were subsequently identified at this resolution using the HOMER program findTADsAndLoops.pl with the following parameters: -res 5000 -minDist 25,000 -maxDist 2,000,000 -poissonLoopLocalBg 0.01 -poissonLoopGlobalBg 1 × 10^−5^. Briefly, -res defines the Hi-C map resolution; -minDist and -maxDist set the interaction distance range, and -poissonLoopLocalBg and -poissonLoopGlobalBg specify the Poisson *p*-value thresholds for interactions relative to local and global backgrounds, respectively. For the identification of A/B compartments, HOMER recommends a resolution of 25 kb for Hi-C maps generated from 200 million to 1 billion read pairs. Accordingly, A/B compartments were identified at 25-kb resolution using the HOMER program runHiCpca.pl with the parameters -res 25,000 -window 50,000, where -res defines the resolution and -window specifies the overlapping window size.

ChIP-seq peaks for 133, 316, and 265 proteins in GM12878, K562, and HepG2 cell lines, respectively, were downloaded from the NCBI Gene Expression Omnibus under the accession number GSE104247 (accessed on 18 May 2023) [16] and from the UCSC Genome Browser database (http://hgdownload.soe.ucsc.edu/goldenPath/hg19/database/) (accessed on 20 June 2023). All peak coordinates were aligned to the hg19 genome assembly, and peaks from multiple replicate files across different laboratories were merged using a union strategy. For each protein, peaks from all replicate files were combined into a single file and sorted by chromosome and start position. Peaks with at least 1 bp of overlap were subsequently merged into a single continuous region spanning the full extent of all merged peaks.

Aligned ChIP-seq reads for control samples and ten histone modifications (H3K4me1, H3K4me2, H3K4me3, H3K9ac, H3K27ac, H3K79me2, H3K36me3, H4K20me1, H3K9me3, and H3K27me3) in GM12878, K562, and HepG2 cell lines were downloaded from the UCSC Genome Browser database (http://hgdownload.soe.ucsc.edu/goldenPath/hg19/encodeDCC/wgEncodeBroadHistone/) (accessed on 10 September 2025). For each enhancer or promoter, the modification level for each histone mark was determined by counting filtered reads overlapping the region.

Aligned paired-end RNA-seq reads for GM12878, K562, and HepG2 cell lines [17] were also downloaded from the UCSC Genome Browser database (http://hgdownload.soe.ucsc.edu/goldenPath/hg19/encodeDCC/wgEncodeCaltechRnaSeq/) (accessed on 5 December 2024). Expression levels for all UCSC genes were quantified using Cufflinks (v 2.2.1) [18] with default parameters.

### 2.2. Classification of Activators and Repressors

A total of 2164 human activators and 1144 repressors were retrieved from the UniProt Knowledgebase (http://www.uniprot.org/) (accessed on 14 July 2025) [19] using the search terms “activator AND (organism_id:9606) AND (reviewed:true)” and “repressor AND (organism_id:9606) AND (reviewed:true)”, respectively. According to the dataset, each protein located within enhancer regions was annotated as an activator, repressor, dual-function protein, or other.

### 2.3. Enrichment of Histone Modifications Within Enhancer or Promoter Regions

For each histone modification, aligned reads overlapping enhancer or promoter regions were quantified. A region was considered enriched for a histone modification if it satisfied two criteria: a read count exceeding a threshold estimated from a background model and a fold change greater than 2 over the control.

In the background model, the threshold *t* for each region was determined based on both the total number of mapped reads and the region length. Specifically, *t* was defined as the smallest integer satisfying *P*(*X* > *t*) < 10^−4^, where *X* follows a Poisson distribution with the mean parameter set to the expected number of reads mapped to the region by random chance [20], which was calculated as:

$$Expected\;reads=\frac{Number\;of\;mapped\;reads\times Region\;length}{Genome\;size\times 0.9}$$

where Genome size × 0.9 represents the effective genome size, defined as the fraction of the human genome that can be uniquely mapped by sequencing reads.

Histone modification enrichment was further validated by calculating the fold change relative to control read counts in the same region, and regions with a fold change greater than 2 were considered enriched [21]. The fold change was calculated as:

$$\mathrm{Fold}\;\mathrm{change}=\frac{ChIP\;reads}{Control\;reads}\times \frac{Total\;control\;reads}{Total\;ChIP\;reads}$$

### 2.4. Identification of E-P Loops

The identification of E-P loops was based on the genomic overlap between loop anchors and known promoter/enhancer annotations. An E-P loop was defined as a chromatin loop with one anchor overlapping an enhancer and the other overlapping a promoter by at least 1 bp.

## 3. Results

### 3.1. Identification of Enhancer-Associated Chromatin Loops

**Figure 1:**
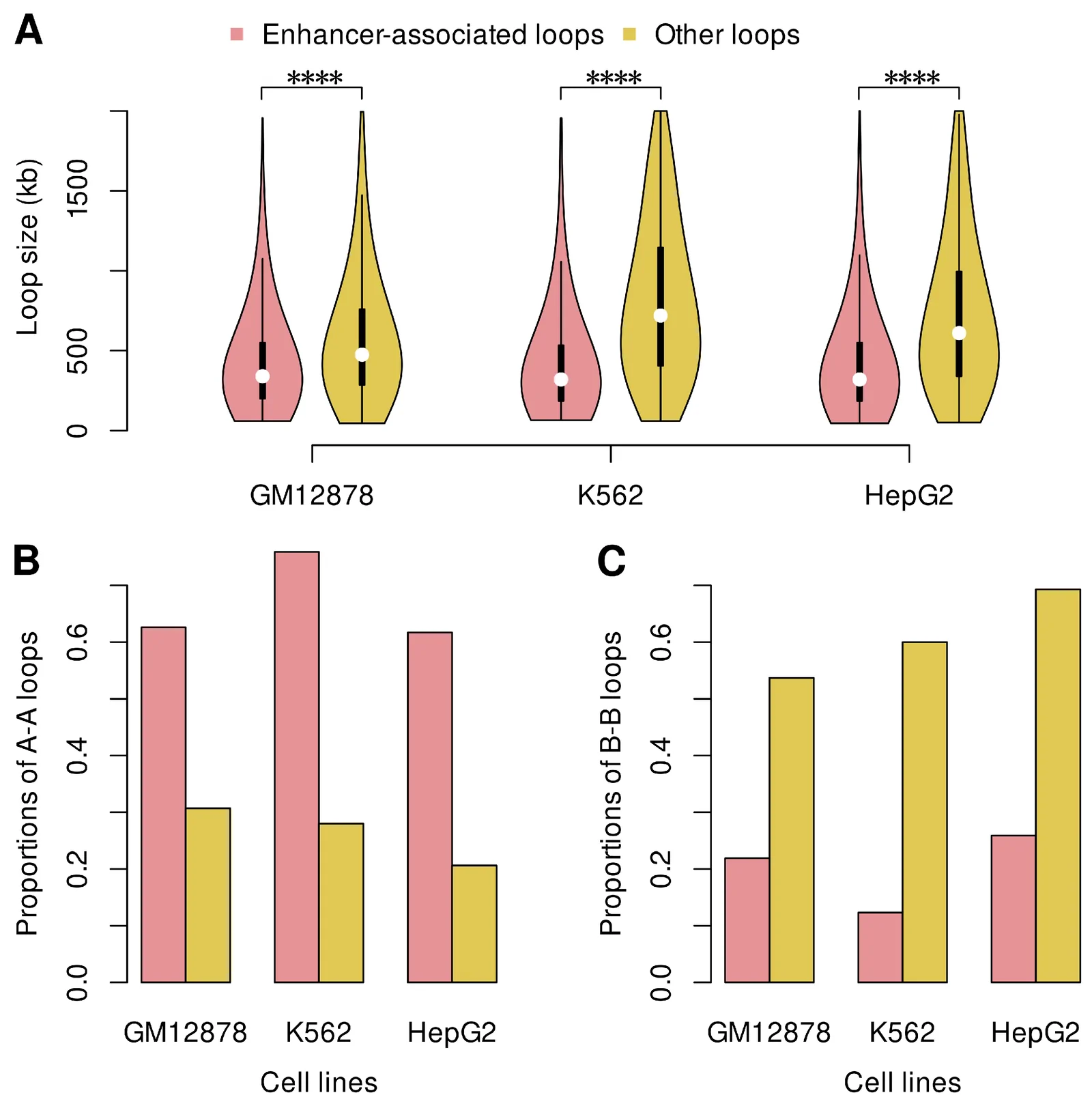
Enhancer-associated chromatin loops are relatively small and localize primarily to the A compartment. (**A**) The size distribution of chromatin loops in GM12878, K562, and HepG2 cells. In each violin plot, the white dot represents the median, and the thick black line indicates the interquartile range. (**B**,**C**) Proportions of A-A and B-B loops in GM12878, K562, and HepG2 cells. **** represents a *p*-value of less than 2.2 × 10^−16^.

A total of 4802, 6987, and 9334 chromatin loops (Table S1) were identified at 5-kb resolution in GM12878, K562, and HepG2 cells, respectively, among which 1399, 1394, and 4063 loops (Table S2) contained anchors overlapping enhancers and were therefore designated as enhancer-associated. The median size of these enhancer-associated loops was 340, 320, and 320 kb in GM12878, K562, and HepG2 cells, respectively, which was significantly smaller than that of other loops (Figure 1A; Wilcoxon rank-sum test, *p* < 2.2 × 10^−16^). Besides, the proportions of enhancer-associated loops classified as A-A loops (both anchors in the A compartment) were 62.6%, 75.9%, and 61.7% in GM12878, K562, and HepG2 cells, respectively, whereas B-B loops (both anchors in the B compartment) accounted for 21.9%, 12.3%, and 25.9% (Figure 1B,C). This distribution was opposite to that observed for other loops, pointing to an active role for enhancers in transcriptional regulation. Fine-scale compartment analysis at 500-bp resolution revealed that active enhancers and promoters may localize to A compartment regions surrounded by B compartment intervals [9]. Given that averaging interaction profiles from neighboring loci at 25-kb resolution would overlook such fine-scale features, we speculate that more enhancer-associated loops would be identified as A-A loops at higher resolution.

### 3.2. Protein Co-Localization at Loop-Anchored Enhancers

**Figure 2:**
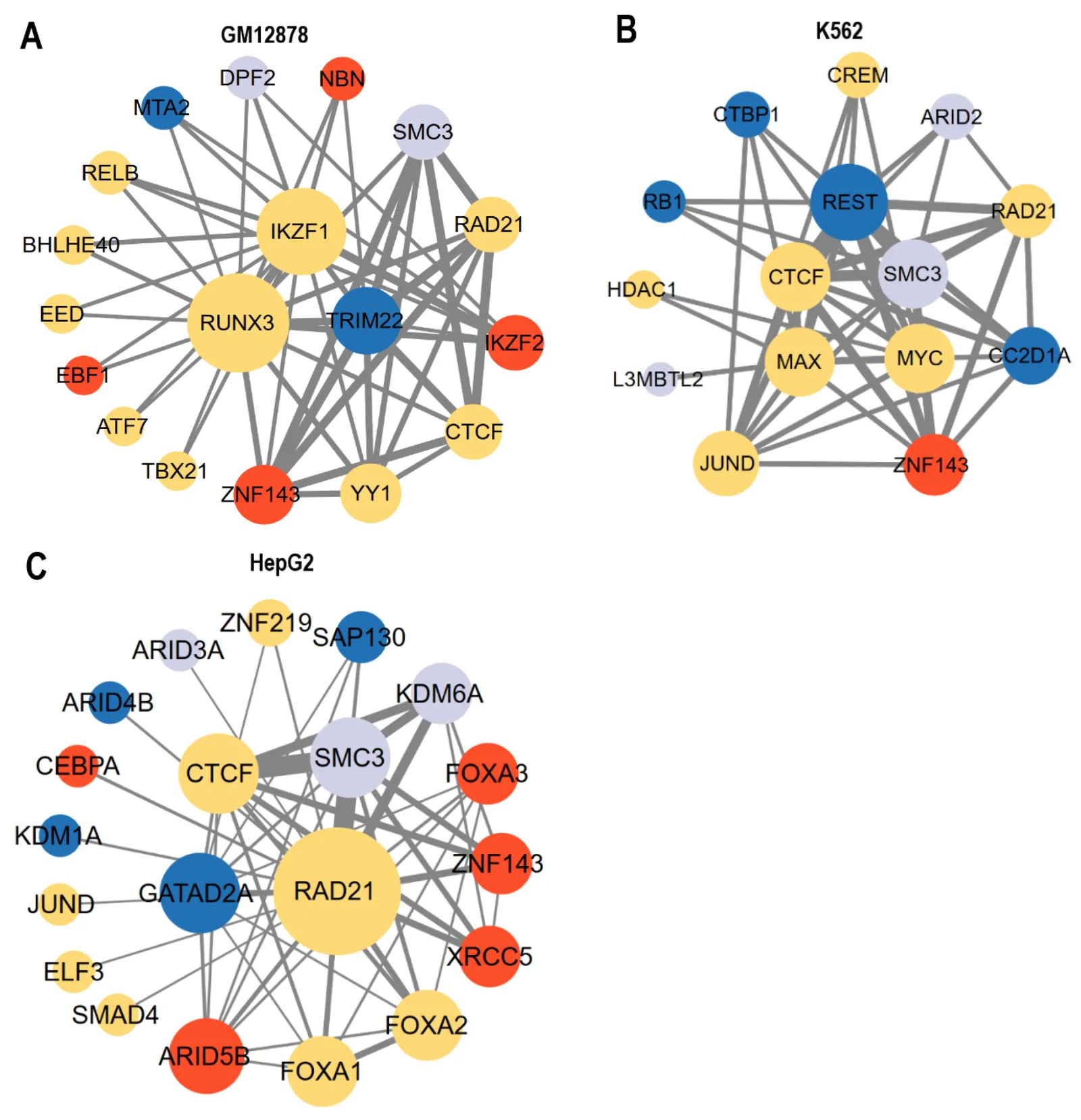
Protein co-localization at loop-anchored enhancers in GM12878 (**A**), K562 (**B**), and HepG2 (**C**) cells. Nodes are colored in red, blue, gold, and grey to represent activators, repressors, dual-function proteins, and other proteins, respectively. Node size scales with degree centrality, and edge width scales with the co-occurrence frequency of protein pairs.

In order to characterize the co-localization patterns of proteins at loop-anchored enhancers, we calculated the co-localization frequency of each pair of proteins at these enhancer regions, and visualized the top 50 pairs with the highest frequencies using Cytoscape (v 3.10.0) [22]. Generally, dual-function proteins with both activating and repressing activities were found to localize more frequently at loop-anchored enhancers (Figure 2), suggesting that they may modulate enhancer activity in a context-dependent manner to precisely regulate the expression of target genes. Specifically, in GM12878, K562, and HepG2 cells, the proteins that most frequently co-localized with other proteins at loop-anchored enhancers were Runt-related transcription factor 3 (RUNX3), RE1-silencing transcription factor (REST), and RAD21, respectively. RUNX3 functions as a lineage-restricted transcription factor and plays a crucial role in mediating chromatin loop formation. RUNX3 has been shown to bind to enhancers and bridge them with promoters, thereby facilitating the expression of target genes through the formation of a RUNX3-containing activator complex at the restriction point [23]. REST binds to RE1 sites and recruits corepressor complexes, which in turn promote heterochromatin formation and facilitate the spatial proximity between distal silencer elements and target genes. The combination of a REST inhibitor and an EZH2 inhibitor has been shown to act synergistically to cause a severe loss of chromatin loops [24]. RAD21 is a core subunit of the cohesin complex, which forms a ring-like structure that dynamically extrudes DNA to form chromatin loops [4].

Although the protein co-localization patterns varied among cell types, CTCF, RAD21, SMC3, and ZNF143 consistently and frequently co-localized with one another at loop-anchored enhancers across all three cell types (Figure 2). In the loop extrusion model, the cohesin complex (comprising SMC1, SMC3, RAD21, and STAG1/2) functions as a motor that drives the process of DNA loop extrusion, whereas CTCF acts as a boundary element that stalls cohesin and anchors loop ends, particularly at convergently oriented binding sites [5]. ZNF143 further regulates these loops by serving as a cofactor of the CTCF-cohesin complex [25]. In GM12878, K562, and HepG2 cells, we found that 21.6%, 23.4%, and 22.2% of enhancer-associated loops, respectively, contained convergent CTCF binding sites at both anchors, supporting their formation via the loop extrusion model.

### 3.3. Histone Modifications at Loop-Anchored Enhancers

**Figure 3:**
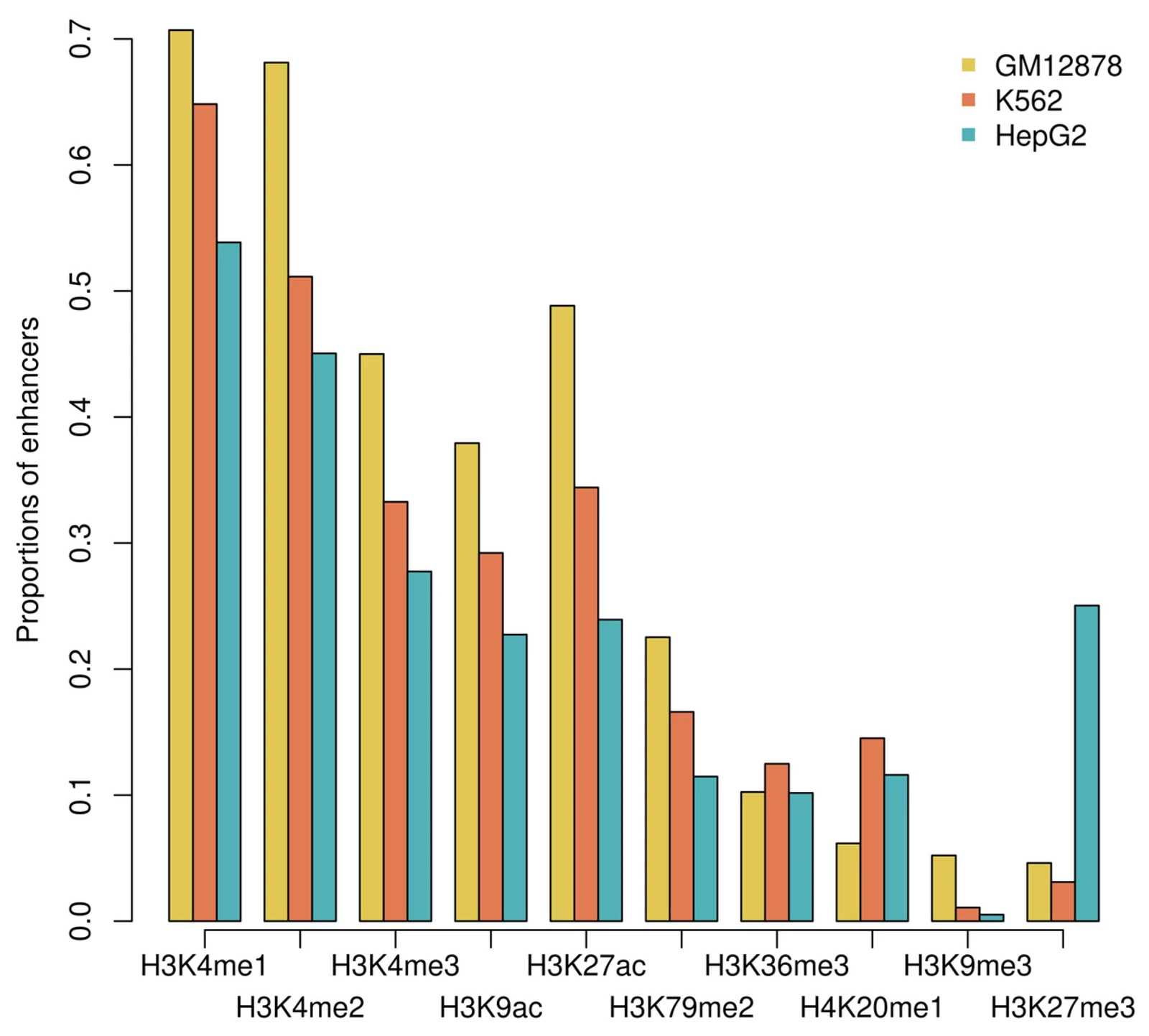
Enrichment of histone modifications at loop-anchored enhancers in GM12878, K562, and HepG2 cells.

We investigated the enrichment of individual histone modifications at loop-anchored enhancers, and found that H3K4me1 was the most enriched histone modification. In GM12878, K562, and HepG2 cells, 70.7%, 64.8%, and 53.9% of loop-anchored enhancers were enriched for H3K4me1 (Figure 3), respectively, consistent with its role as a marker for both active and poised enhancers [2]. Notably, 42.1%, 28.8%, and 19.8% of loop-anchored enhancers exhibited co-enrichment of H3K4me1 and H3K27ac in the absence of H3K27me3, respectively, suggesting their active status. Although loop-anchored enhancers were generally enriched for active histone modifications, the repressive mark H3K27me3 was particularly prominent in HepG2 cells. In HepG2 cells, 25.0% of loop-anchored enhancers showed H3K27me3 enrichment, and 9.7% exhibited co-enrichment of H3K4me1 and H3K27me3 in the absence of H3K27ac. Such co-enriched enhancers mark a poised state, and may be associated with developmental genes, as well as genes that can be rapidly activated upon specific signals or stress [26].

**Figure 4:**
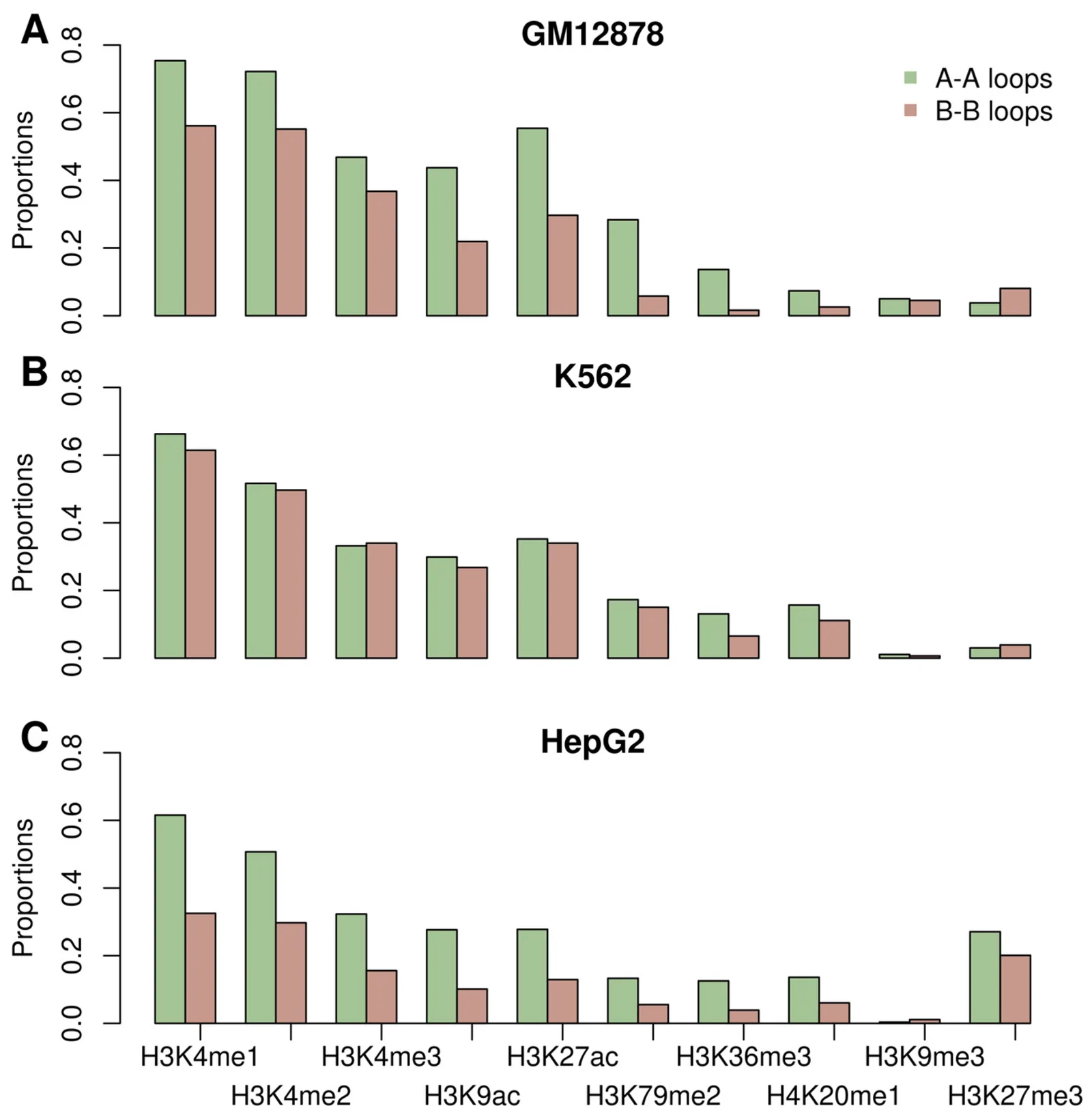
Enrichment of histone modifications at enhancers anchored in A-A and B-B loops in GM12878 (**A**), K562 (**B**), and HepG2 (**C**) cells.

We further categorized enhancers based on whether they were anchored in A-A or B-B loops and examined histone modification enrichment in the two groups. Compared to enhancers anchored in B-B loops, those anchored in A-A loops showed greater enrichment of four active histone modifications (H3K4me1, H3K4me2, H3K9ac, and H3K27ac) across all three cell lines (Figure 4). Specifically, the proportions of H3K4me1-enriched enhancers in A-A loops exceeded those in B-B loops by 19.2%, 4.8%, and 29.0% in GM12878, K562, and HepG2 cells, respectively, whereas the corresponding differences for H3K27ac were 25.7%, 1.2%, and 14.9%. Furthermore, in GM12878 and K562 cells, enhancers anchored in A-A loops showed lower H3K27me3 enrichment, with the proportions of H3K27me3-enriched enhancers decreased by 4.3% and 0.9%, respectively. These results suggest that enhancers anchored in A-A loops tend to be in an active state, whereas those in B-B loops tend to be in a poised or repressed state.

### 3.4. Expression of Target Genes Associated with E-P Loops

**Figure 5:**
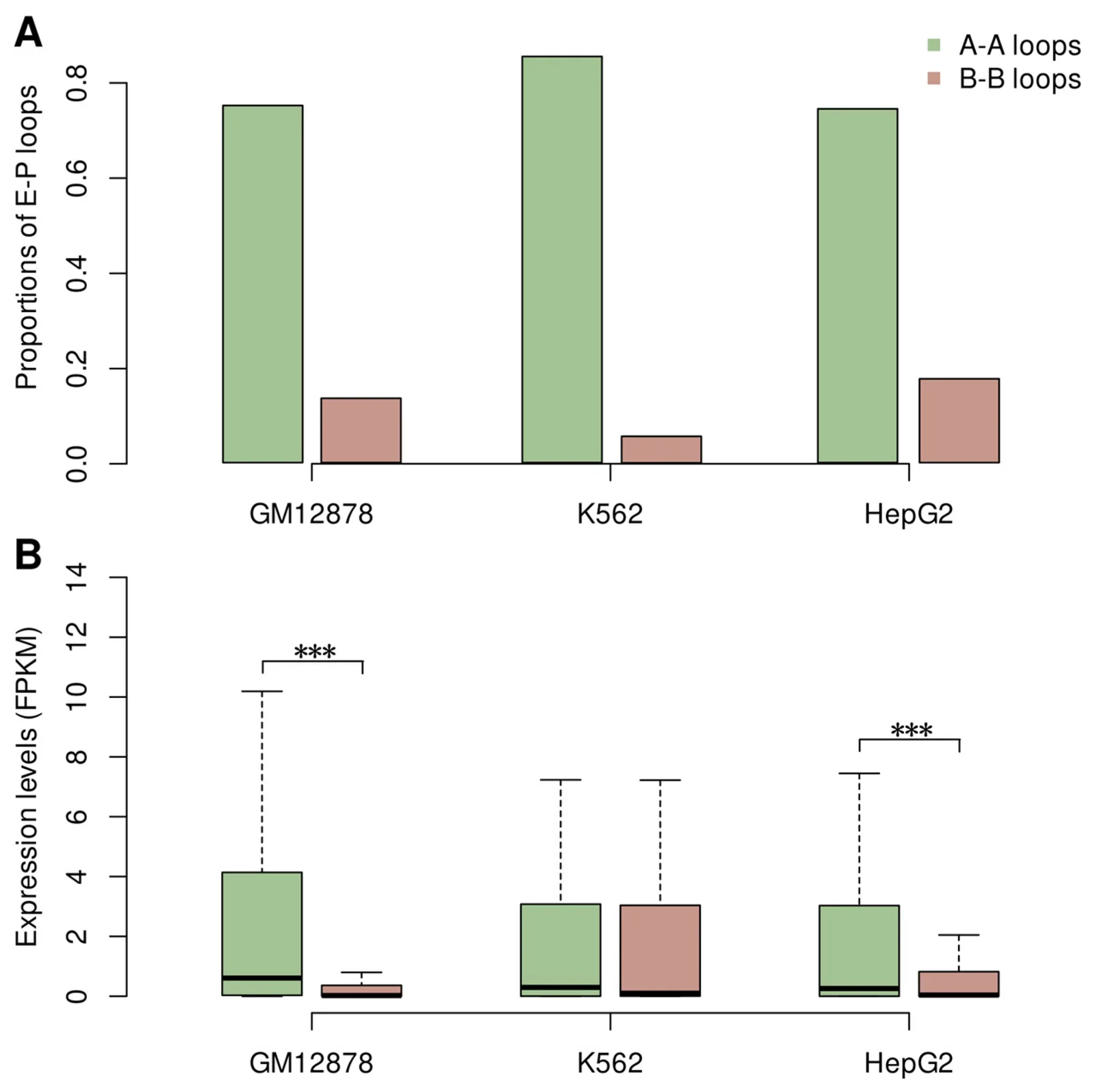
A-A E-P loops are associated with higher target gene expression than B-B loops. (**A**) Proportions of A-A and B-B E-P loops in GM12878, K562, and HepG2 cells. (**B**) Expression levels of target genes associated with A-A and B-B E-P loops in GM12878, K562, and HepG2 cells. *** represents a *p*-value of less than 1.5 × 10^−8^.

To investigate how enhancers regulate their target genes, 260, 266, and 732 E-P loops (Table S3) were identified in GM12878, K562, and HepG2 cells, respectively, of which 75.0%, 85.3%, and 74.3% were A-A loops (Figure 5A). Quantitative RNA-seq analysis revealed that A-A E-P loops were associated with significantly higher target gene expression than B-B loops in both GM12878 and HepG2 cells (Figure 5B; Wilcoxon rank-sum test, *p* < 1.5 × 10^−8^). In K562 cells, however, the two loop types showed no significant difference in target gene expression, suggesting that the formation of E-P loops within A compartments alone may not be sufficient to drive active expression of target genes, and instead, that additional regulatory factors may be required.

**Figure 6:**
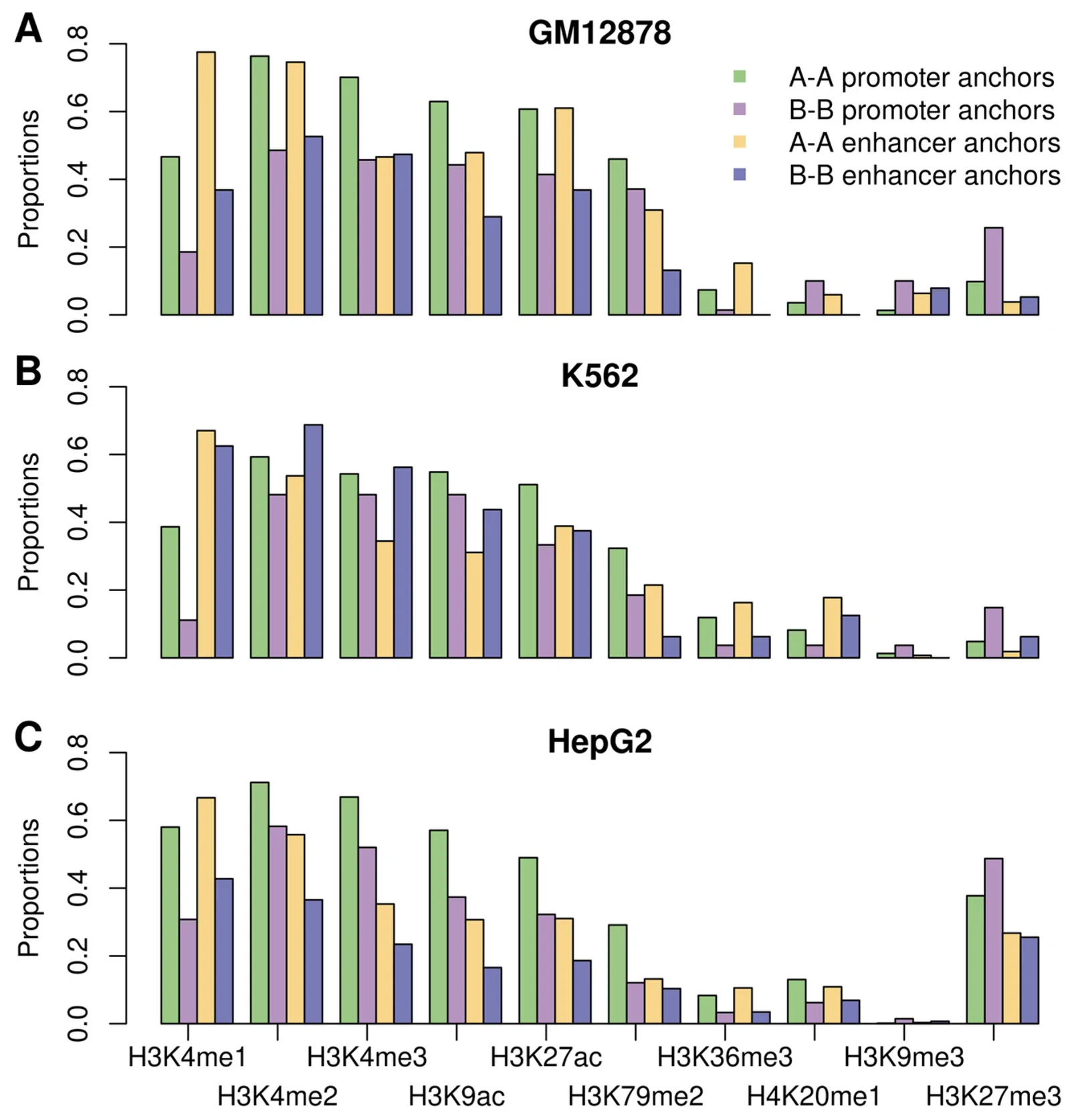
Enrichment of histone modifications at A-A and B-B E-P loop anchors in GM12878 (**A**), K562 (**B**), and HepG2 (**C**) cells.

We further investigated histone modification enrichment at both anchors of A-A and B-B E-P loops. At promoter anchors, A-A E-P loops exhibited greater enrichment of H3K4me1, H3K4me2, H3K4me3, H3K9ac, and H3K27ac, along with lower enrichment of H3K27me3, than B-B loops in GM12878, K562, and HepG2 cells (Figure 6). However, the patterns at enhancer anchors varied across these cell lines. In GM12878 and HepG2 cells, the proportions of H3K4me1-enriched enhancers in A-A E-P loops exceeded those in B-B loops by 40.7% and 23.9%, respectively, with corresponding differences of 24.2% and 12.4% for H3K27ac. In K562 cells, however, these differences were substantially smaller: 4.5% for H3K4me1 and 1.4% for H3K27ac. For H3K4me2 and H3K9ac, enhancer anchors of A-A E-P loops again showed higher proportions than their B-B counterparts in GM12878 and HepG2 cells (by 21.9% and 19.2% for H3K4me2, and 18.9% and 14.1% for H3K9ac, respectively). In K562 cells, by contrast, the trend was reversed: enhancer anchors of B-B E-P loops exceeded their A-A counterparts by 15.0% for H3K4me2 and 12.6% for H3K9ac. These results suggest that in K562 cells, the gain of active histone marks at enhancer anchors of B-B E-P loops may modulate enhancer activity and consequently affect the expression of target genes. Indeed, in K562 cells, the *HLX* gene, involved in myeloid development [27], was enriched for H3K4me1, H3K4me2, H3K9ac, and H3K27ac at its enhancer region, whereas the *COX10* gene, linked to tumor growth and progression in various cancers [28], was enriched for H3K4me2 and H3K9ac. Both genes were associated with B-B E-P loops and exhibited expression levels above the third quantile of all genes in K562 cells.

**Figure 7:**
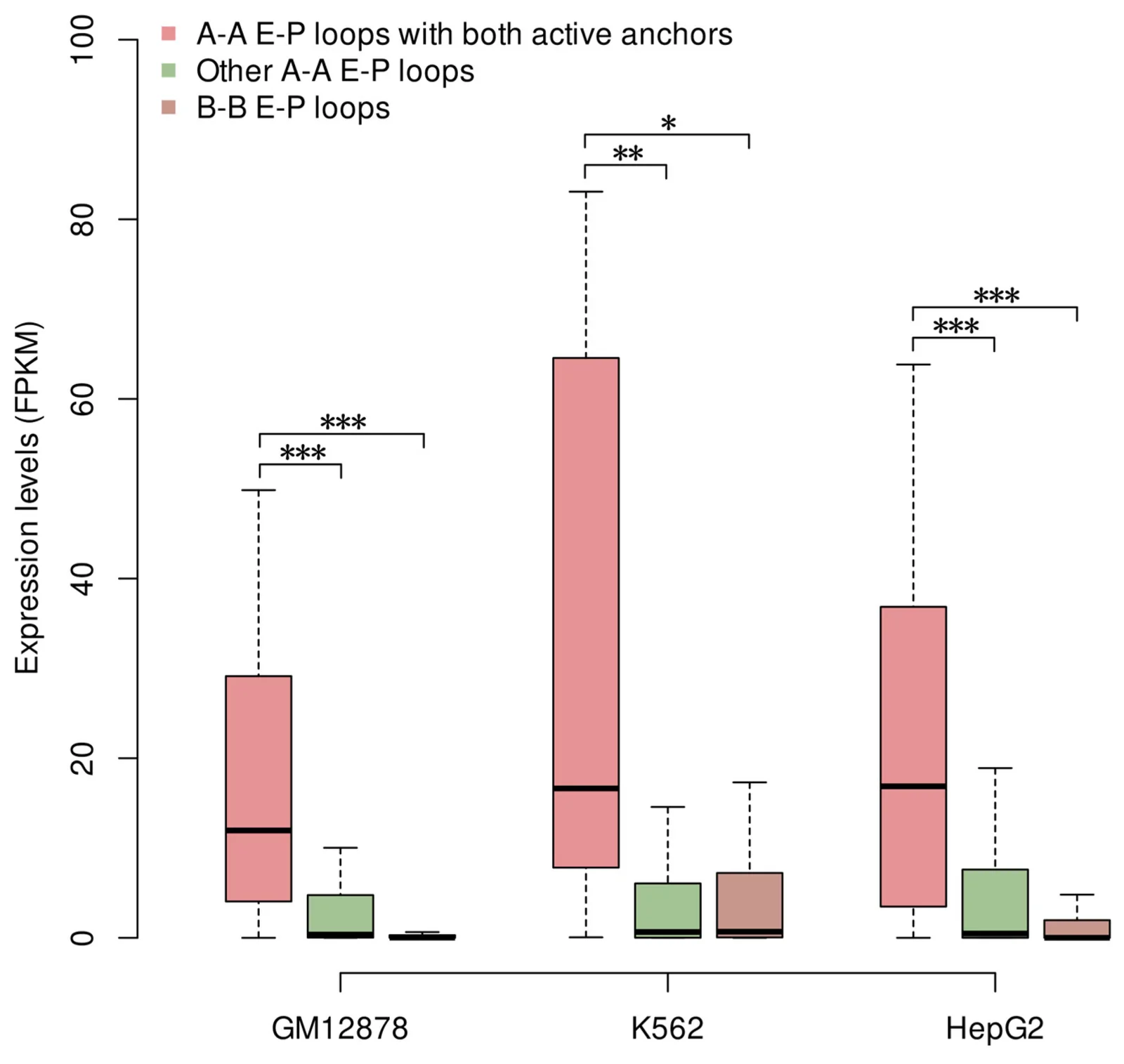
A-A E-P loops with both active anchors exhibit higher target gene expression. *, **, and *** represent *p*-values of less than 3.4 × 10^−4^, 1.3 × 10^−6^, and 1.5 × 10^−8^, respectively.

We considered enhancers as active if they showed co-enrichment of H3K4me1 and H3K27ac without H3K27me3, and promoters as active if they were enriched for H3K4me3 in the absence of H3K27me3 [29]. Based on these definitions, we further classified A-A E-P loops into those with both active anchors and others, and found that loops with dual active anchors exhibited significantly higher target gene expression than other A-A loops in GM12878, K562, and HepG2 cells (Figure 7; Wilcoxon rank-sum test, *p* < 1.3 × 10^−6^). Similarly, these dual-active A-A loops also exhibited higher target gene expression than B-B E-P loops across all three cell lines (Figure 7; Wilcoxon rank-sum test, *p* < 3.4 × 10^−4^). These results suggest that active histone modifications at both enhancers and promoters may be necessary for enhancer activity and elevated target gene expression.

## 4. Discussion

Although enhancers can reside millions of base pairs away from their target genes, our analysis revealed that enhancer-associated loops were relatively small compared to other loops, spanning mainly hundreds of kilobases. Chromatin loops were identified using HOMER within a restricted size range of 25 kb to 2 Mb, which may bias the size distribution. However, the small proportion of loops outside this range suggests that such bias is likely minor. This interpretation is supported by previous findings showing that fewer than 5% of chromatin loops are shorter than 50 kb or longer than 2 Mb in GM12878 and K562 cells [12,30]. We further found that the enhancers anchoring chromatin loops were frequently bound by proteins annotated as dual-function in the UniProt Knowledgebase, suggesting a potential role in modulating enhancer activity in a context-dependent manner. It should be noted that such a global dual-function annotation does not guarantee that both activities are exerted at the specific enhancer loci analyzed in our study, as protein activity is highly dependent on the local regulatory context. Specifically, CTCF, RAD21, SMC3, and ZNF143, which cooperate in loop extrusion, frequently co-localized with one another at loop-anchored enhancers. However, approximately one-fifth of enhancer-associated loops contained convergent CTCF binding sites at both anchors, suggesting that the majority of these loops may not be formed through CTCF-dependent loop extrusion. We speculate that liquid–liquid phase separation, driven by multivalent interactions between protein IDRs, could represent an alternative mechanism that brings distal regulatory elements to target genes via the formation of transcriptional condensates [7]. According to the annotation of protein IDRs in the MobiDB database [31], all proteins frequently co-localized at loop-anchored enhancers (Figure 2) contained IDRs, raising the possibility that phase separation may contribute to loop formation. Nevertheless, direct experimental approaches are required to further validate the role of these protein-binding events in chromatin looping.

The majority of enhancer-associated loops were A-A loops. Enhancers anchored in A-A loops showed greater enrichment of active histone modifications and lower enrichment of H3K27me3 than those anchored in B-B loops, suggesting that A-A loop-anchored enhancers tend to be in an active state. However, in K562 cells, enhancers anchored in B-B loops exhibited minor changes in active histone modification enrichment compared to their A-A counterparts (Figure 4), and even showed higher proportions of H3K4me2 and H3K9ac enrichment for E-P loops (Figure 6), which may contribute to the higher expression of a subset of genes associated with B-B loops. Consistent with this, the *HLX* and *COX10* genes, both associated with B-B E-P loops, showed enrichment for active histone modifications at their enhancer regions and higher expression levels. Notably, this cell-type-specific anomaly may result from the resolution constraints of A/B compartment identification. At higher resolution, these active enhancers and promoters currently located in B compartments might be reclassified as A compartments. This possibility is supported by a previous study showing that active regulatory elements frequently reside within small A compartment islands surrounded by broader B compartment intervals [9].

In K562 cells, A-A loop-associated genes showed comparable expression levels to B-B loop genes overall. However, A-A E-P loops with both active anchors exhibited significantly higher target gene expression than other A-A loops and B-B loops, consistent with observations in GM12878 and HepG2 cells. These results suggest that enrichment of the active histone modifications H3K4me1 and H3K27ac at enhancers is associated with enhancer activity, and in combination with chromatin interactions in the A compartment, may contribute to higher target gene expression. Multi-bromodomain proteins, including the transcriptional regulator BRD4, have been reported to induce the formation of distinct droplets from highly acetylated chromatin [32]. These droplets are immiscible with unmodified chromatin droplets and may serve to facilitate E-P communication and promote gene expression. In the present study, active enhancers were defined as those enriched for H3K4me1 and H3K27ac in the absence of H3K27me3. Although this co-enrichment is well recognized as a marker for active enhancers, other histone modifications, such as H3K4me2 and H3K9ac [2,33], may also modulate enhancer activity. However, these modifications were not examined in our analysis.

It is estimated that there are hundreds of thousands of putative enhancers in the human genome [34]. Our study analyzed tens of thousands of enhancers, of which 3.4%, 3.7%, and 8.9% were anchored in chromatin loops in GM12878, K562, and HepG2 cells, respectively. These small proportions may be explained by three factors. First, chromatin loops shorter than 25 kb or longer than 2 Mb were excluded from our analysis. In HOMER, the default minimum interaction distance for identifying significant chromatin loops is set to 25 kb [15], as interactions shorter than this threshold are difficult to distinguish from baseline polymer dynamics, random looping fluctuations, and general chromatin compaction. Conversely, the maximum distance is set to 2 Mb, because the vast majority of biologically functional, cohesin-driven loops fall within this range, and this threshold balances computational efficiency with biological relevance. Second, the application of more stringent parameters (-poissonLoopLocalBg and -poissonLoopGlobalBg) resulted in the identification of relatively fewer chromatin loops. Specifically, setting these parameters to 0.05 and 0.001 yielded 11,457, 17,626, and 19,686 chromatin loops in GM12878, K562, and HepG2 cells, respectively. Third, standard Hi-C generates sparse and noisy contact maps and lacks sufficient sequencing depth and resolution to accurately identify dynamic, highly specific enhancer-associated loops. In contrast, advanced chromosome conformation capture techniques, such as Micro-C [35], provide nucleosome-level resolution and superior signal-to-noise ratios, facilitating the capture of short-range loops and multi-enhancer hubs [36] to reveal how enhancers regulate target gene transcription.

## 5. Conclusions

In this study, we identified enhancer-associated chromatin loops and examined the co-localization patterns of proteins and the enrichment of histone modifications at loop-anchored enhancers in GM12878, K562, and HepG2 cell lines. We found that enhancer-associated chromatin loops were relatively small in size and predominantly A-A loops. These loop-anchored enhancers were frequently bound by dual-function proteins, and were primarily enriched for H3K4me1. Further classification of enhancers anchored in A-A versus B-B loops revealed that A-A loop-anchored enhancers exhibited greater enrichment of active histone modifications (H3K4me1, H3K4me2, H3K9ac, and H3K27ac) and lower H3K27me3 enrichment than their B-B loop-anchored counterparts. Moreover, analysis of E-P loop target genes demonstrated that A-A loops with active enhancer and promoter anchors were associated with significantly higher target gene expression than other A-A loops and B-B loops. Together, these results suggest that dual-function proteins may cooperate with histone modifications to modulate enhancer activity, promote chromatin looping, and ultimately regulate transcription.

## Data Availability

The original contributions presented in this study are included in the article/Supplementary Materials. Further inquiries can be directed to the corresponding author.

## Supplementary Materials

The following supporting information can be downloaded at: https://www.mdpi.com/article/10.3390/biology15161331/s1, Table S1: Genomic coordinates of chromatin loops identified by HOMER in GM12878, K562, and HepG2 cells; Table S2: Genomic coordinates and loop types of enhancer-associated chromatin loops in GM12878, K562, and HepG2 cells; Table S3: Enhancer–promoter loop features in GM12878, K562, and HepG2 cells.

## Abbreviations

The following abbreviations are used in this manuscript:

E-P	enhancer–promoter
CTCF	CCCTC-binding factor
IDR	intrinsically disordered regions
kb	kilobase
RUNX3	Runt-related transcription factor 3
REST	RE1-silencing transcription factor
